# Generation of macro- and microplastic databases by high-throughput FTIR analysis with microplate readers

**DOI:** 10.1007/s00216-024-05127-w

**Published:** 2024-01-13

**Authors:** Win Cowger, Lisa Roscher, Hannah Jebens, Ali Chamas, Benjamin D. Maurer, Lukas Gehrke, Gunnar Gerdts, Sebastian Primpke

**Affiliations:** 1 Moore Institute for Plastic Pollution Research, Long Beach, CA USA; 2 Alfred Wegener Institute Helmholtz Centre for Polar and Marine Research, Helgoland, Germany; 3 University of California, Riverside, CA USA; 4 National Renewable Energy Laboratory, Golden, CO USA

**Keywords:** Plastic pollution, Microplastics, FTIR, Database, High-throughput, Spectroscopy

## Abstract

FTIR spectral identification is today’s gold standard analytical procedure for plastic pollution material characterization. High-throughput FTIR techniques have been advanced for small microplastics (10–500 µm) but less so for large microplastics (500–5 mm) and macroplastics (> 5 mm). These larger plastics are typically analyzed using ATR, which is highly manual and can sometimes destroy particles of interest. Furthermore, spectral libraries are often inadequate due to the limited variety of reference materials and spectral collection modes, resulting from expensive spectral data collection. We advance a new high-throughput technique to remedy these problems using FTIR microplate readers for measuring large particles (> 500 µm). We created a new reference database of over 6000 spectra for transmission, ATR, and reflection spectral collection modes with over 600 plastic, organic, and mineral reference materials relevant to plastic pollution research. We also streamline future analysis in microplate readers by creating a new particle holder for transmission measurements using off-the-shelf parts and fabricating a nonplastic 96-well microplate for storing particles. We determined that particles should be presented to microplate readers as thin as possible due to thick particles causing poor-quality spectra and identifications. We validated the new database using Open Specy and demonstrated that additional transmission and reflection spectra reference data were needed in spectral libraries.

**Graphical abstract:**

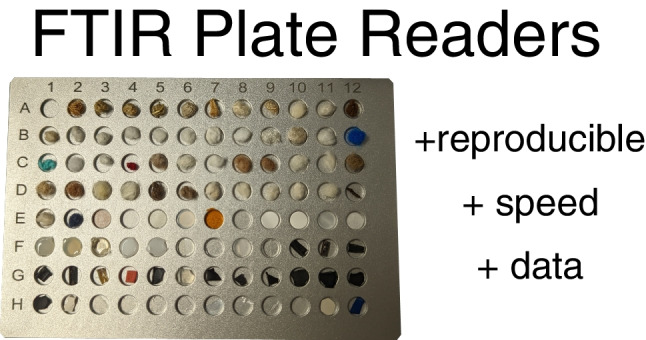

**Supplementary Information:**

The online version contains supplementary material available at 10.1007/s00216-024-05127-w.

## Introduction

Fourier transform infrared (FTIR) spectroscopy is currently a gold standard procedure for the material characterization of microplastic (1–5000 µm) particles [1–3]. FTIR spectroscopy is a non-destructive technique that provides rich information about chemical bonds in materials and can accurately differentiate plastics from nonplastics [4, 5]. High-throughput spectroscopy techniques like mapping FTIR are now gaining widespread use [6, 7]. These techniques have improved sample throughput by orders of magnitude and made plastic particles in the nanometer range possible to characterize [8]. However, the optimal particle size range for the most widely used high-throughput FTIR technique (microscope-FTIR) is 10–500 µm, and there does not currently exist a proposed technique for high-throughput FTIR analysis of large microplastic particles (500 µm to 5 mm), sometimes referred to as mesoplastics [9], or macroplastic particles (> 5 mm). These larger particles often comprise most of the plastic mass in many samples [10, 11]. They can also be highly abundant [12], leading to a significant amount of time in manual spectral characterization. Standard attenuated total reflection (ATR) measurement techniques for these larger particles require a long time [13] (in our case, an average of 10 min per particle) to collect a quality particle spectrum, which must be redone if anything goes wrong (e.g., unaligned detector) during spectral collection. This is because the spectroscopist must manually focus the ATR on every particle individually and stay with the device while it collects the spectra. FTIR plate readers allow a spectroscopist to load many (typically 96) particles for high-quality measurement into a single instrument run, thus eliminating manual focusing, standardizing, simplifying, and speeding up data collection. FTIR plate readers have been used extensively to characterize samples in high-throughput (< 1 min per particle) in biology [14–17] and soil research [18, 19] but have not been used to characterize individual particles and are typically used on homogeneous samples. Plate readers have been piloted for plastic pollution research [20] but have not been tested at scale. Our first study goal was to develop a technique for using FTIR plate readers for large microplastic and macroplastic characterization and compare it to ATR.

The lack of reference spectral libraries for reflection and transmission spectral collection modes is one of the largest barriers to utilizing FTIR plate readers in plastic pollution research [13, 21]. Reflection and transmission spectra can differ from the ATR spectra commonly included in commercial and open-source databases [22]. Plastic pollution spectral database development has been a huge challenge even for leading industry spectral database suppliers [23], due to the diversity of the plastic materials [24] and spectra from environmental samples [25]. Plastic spectra can be impacted by particle weathering [26], biofilms [28, 29], and particle orientation (particles are typically presented to spectrometers in a non-homogenized form [30]). Our second goal was to use the high-throughput technique to develop a harmonized database for ATR, reflection, and transmission spectra of relevant materials for studying non-homogenized plastic pollution particles (i.e., plastic, natural organics, and minerals).

## Methods

### Overview

**Fig. 1:**
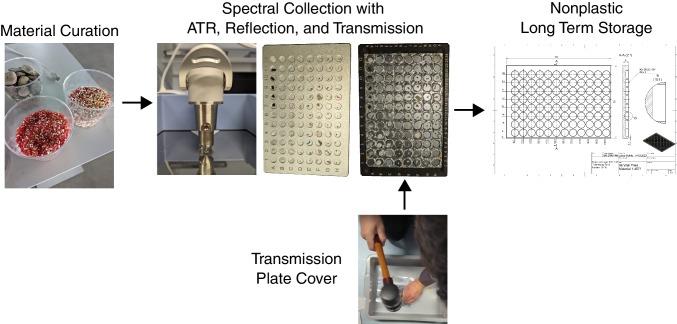
Schematic representation of the major aspects of this study, showing (left to right) the curation of the 637 materials, the high-throughput FTIR processing of individual materials with ATR plus the 96-well plates and fabricated transmission plate cover, and lastly archiving samples with a fabricated nonplastic 96 well plate

The primary goal of this study was to advance the technique of using FTIR plate readers for micro-, meso-, and macroplastic (Fig. 1). To do so, we first curated a large material database of plastic and nonplastic materials. Those materials were analyzed with ATR and an FTIR plate reader for reflection and transmission. The transmission plate would not work without walls due to vibrations in the machine, so we developed a walled cover that could keep the particles from cross-contaminating other wells. We did not have a simple solution for long-term affordable nonplastic storage of the particles, so we developed a 96-well plate that can be fabricated in common machine shops.

### Sample preparation

**Fig. 2:**
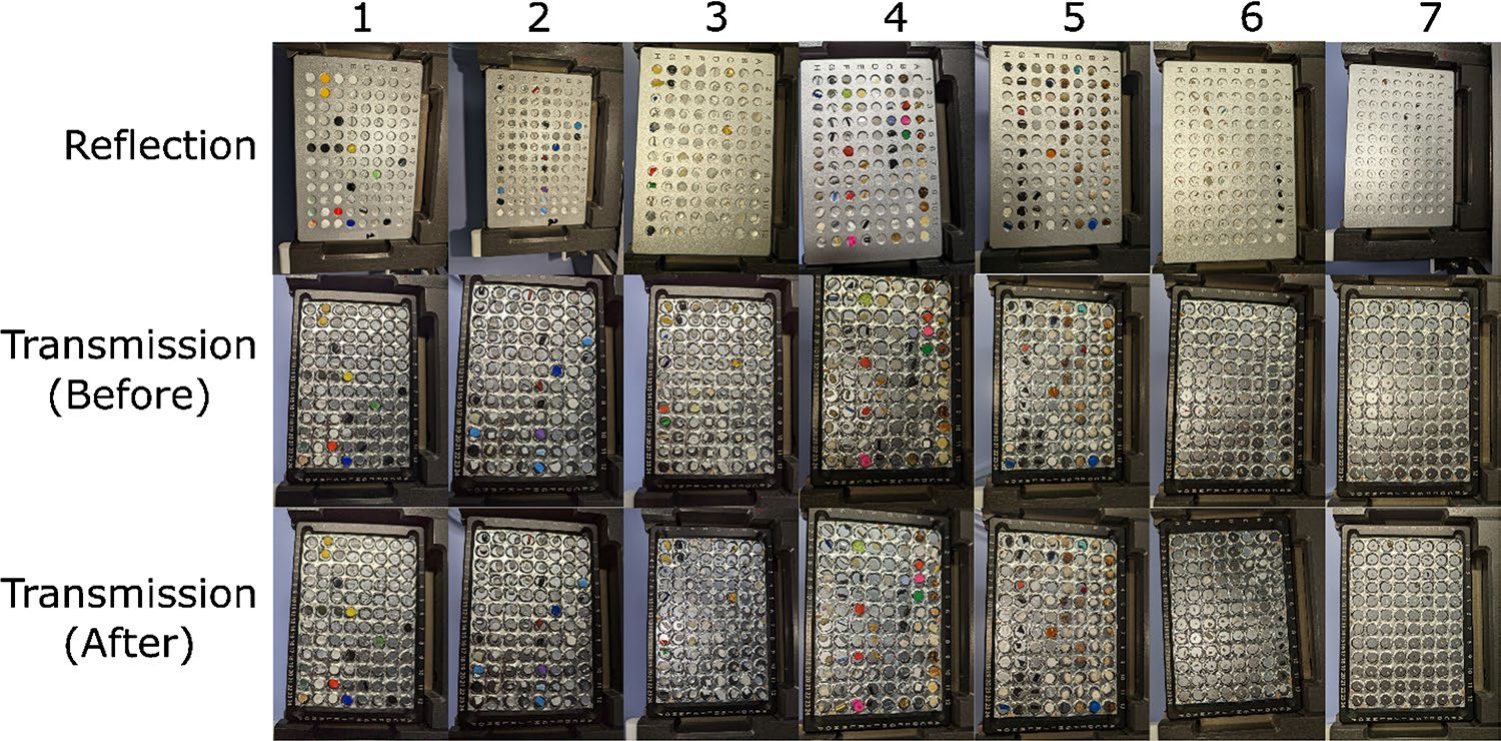
Images of particles in plates for transmission and reflection measurements. Transmission (before) and transmission (after) were compared to assess whether particles moved during the measurement. Each well held a different particle. Spectral collection mode is labeled on the left axis, and the plate number is on the top axis. Transmission plates had a custom-made well overlay from heavy aluminum foil. No particles were observed missing or crossing into another well during the transmission measurement (which can be caused by vibrations in the machine if not using the transmission plate cover)

Particles were collected from the in-house reference standards available in Primpke lab at the Alfred Wegener Institute, the National Renewable Energy Laboratory, the Moore Institute for Plastic Pollution Research, Hawaii Pacific University’s Center for Marine Debris Polymer Kit 1.0 [31], and microplastic samples from environmental samples from Roscher et al. [32, 33]. The standard materials contained 554 plastic materials, 56 natural organic materials (or organics), 3 minerals, 7 other materials, and 31 unknowns, totaling 637 materials. Small particles (< 5 mm) were placed in the well without additional preparation (Fig. 2). Large particles (> 5 mm) were prepared by reducing them to a size that would fit in the 5 mm plate reader wells. Fibrous particles were hand-rolled into small balls (2–5 mm). Ridgid large plastic was clipped using a standard hole punch (3–5 mm) for paper. Film particles were cut with scissors by hand. Pellets were chopped with scissors if they were too large to fit in the well. No granule or liquid particles were assessed with this technique because the transmission plate could not prevent cross-contamination since the wells did not have walls blocking the particles from moving into another well and a large amount of vibration was present in the device, causing particles to shift. A needle was used for extracting and inserting particles that fit snugly in the wells. Contamination is generally less of an issue for mesoplastic and macroplastic because you can see the particles with the naked eye, and particles that large do not shed easily from surfaces. Nevertheless, care was taken to avoid using plastic during sample preparation wherever possible, and plates were cleaned with 99.9% ethanol (Merck, Germany) pre-filtered using 0.2-µm GTTP membranes (Merck, Germany) before measurements or between transfers of one batch of particles to the next. Position A1 was always kept free and used for background measurements.

### Transmission cover creation

**Fig. 3:**
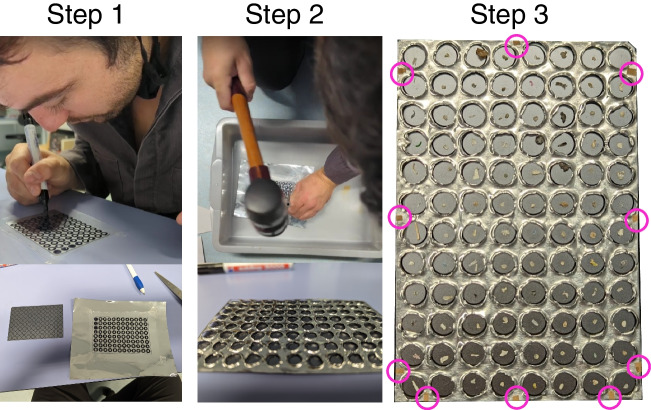
Visual instructions for creating the aluminum overlay for the transmission plates. Step 1: Trace wells and outline of transmission plate on thick plastic and transfer the plastic overlay to a piece of heavy aluminum foil with tape. Step 2: Pound a gaged stamp of the well size with a rubber mallet on top of a hard plastic platform and cut the aluminum to size with scissors. Step 3: Tape the aluminum cover to the silicon plate with small slivers of tape at the edges

The standard transmission plate for the Bruker HTS-XT [34] had a flat surface that could not prevent particle cross-contamination. During the automated movement in the machine, the machine’s vibration of the plate would cause particles to roll into other wells and thus lose their reference in the data. There were other transmission plates with edges on the wells [35], but we were unaware of one that existed for the Bruker HTS-XT. We fabricated an overlay using heavy aluminum foil to prevent particle movement, which we hand-cut using a rubber mallet and a circular hole punch (Fig. 3). We created a template for the hammering by putting a transparent piece of plastic on top of the transmission plate, tracing out where the wells were, and then taping the template to a piece of heavy aluminum foil for cutting (Fig. 3, Step 1). Hammering was done on top of a hard plastic plate to prevent curling of the aluminum when hit and to prevent cutting through the floor. Then, the aluminum foil cutout was flattened by hand to fit tight against the silicon plate (Fig. 3, Step 2). The heavy aluminum foil was then fixed as close to the silicon transmission plate surface as possible using minimal tape (Fig. 3, Step 3). The tape was positioned to avoid overlapping the wells by placing it between them.

### Spectral acquisition parameters

We follow recommendations by Andrade et al. (2020) [30] for minimum information for the publication of infrared spectra in microplastic research. Spectra were collected with a Bruker Tensor 27 with the HTS-XT plate reader attachment (approximately $100 k total cost). The device was flushed with air scrubbed of water and carbon dioxide to prevent atmospheric artifacts. The device used the OPUS software to collect the data. We used the device’s three spectral collection modes, ATR, transmission, and reflection, to collect 1–8 spectra per particle. All particles were assessed with transmission and reflection, but some in plates 3, 4, and 5 were not assessed with ATR due to how time-intensive the ATR data collection was. Six hundred thirthy-seven materials were measured in total, with some replicated in independent wells up to 5 times.

ATR spectra were collected for each particle on two sides of the particles with the diamond ATR attachment of the Tensor 27 with a room temperature detector RT-DLaTGS and a mirror speed of 10 kHz, 32 scans, a 4 wavenumber spectral resolution, from 4000 to 400 wavenumbers, and 6 mm aperture. The background measurement was done before every particle measurement on an open and clean ATR surface and automatically subtracted from the spectra. Fourier transformation was conducted with Mertz phase correction and an apodization function of Blackman–Harris 3-term and 2-zero filling factor. We observed every spectrum collected, and if a particle had drastically different spectra on each side, we noted that. The ATR crystal and tip were cleaned with ethanol between particles.

Transmission spectra were collected with the HTS-XT plate reader using the HTS-XT transmission room temperature detector using a 5-mm aperture, a mirror speed of 10 kHz, 32 scans, and a 4 wavenumber spectral resolution from 4000 to 400 wavenumbers. The background was done before every measurement on an empty transmission well (position A1). Fourier transformation was conducted with Mertz phase correction and an apodization function of Blackman–Harris 3-term and 4-zero filling factor. We tested the impact of changing spectral wavenumber resolution to 8 and collecting only one spectrum per material. We found a high average Pearson correlation between the data sets (0.92), suggesting that changing the parameters slightly to others commonly used does not drastically change the quality of the database produced and that replicates of wells are not strictly mandatory. We also tested the reproducibility of spectral measurements for the same material type cut into different sizes and placed into different wells with different particle orientations. In this test, 5 particle examples were produced from 6 materials: polyethylene film, polyethylene pellets, polypropylene pellets, polypropylene film, polyethylene terephthalate, and tire rubber. Each well was measured 3 times with the ATR and 4 times with reflection and transmission in the plate reader.

Reflection spectra were collected with the HTS-XT plate reader with an LN MCT detector cooled with liquid nitrogen with a 6-mm aperture and a 20-kHz mirror speed, 32 scans, and a 4 wavenumber spectral resolution from 4000 to 620 wavenumbers. Before every measurement, a background measurement was done on the empty reflection plate well (position A1). Fourier transformation was conducted with Mertz phase correction and an apodization function of Blackman–Harris 3-term and 4-zero filling factor.

### Long-term storage

**Fig. 4:**
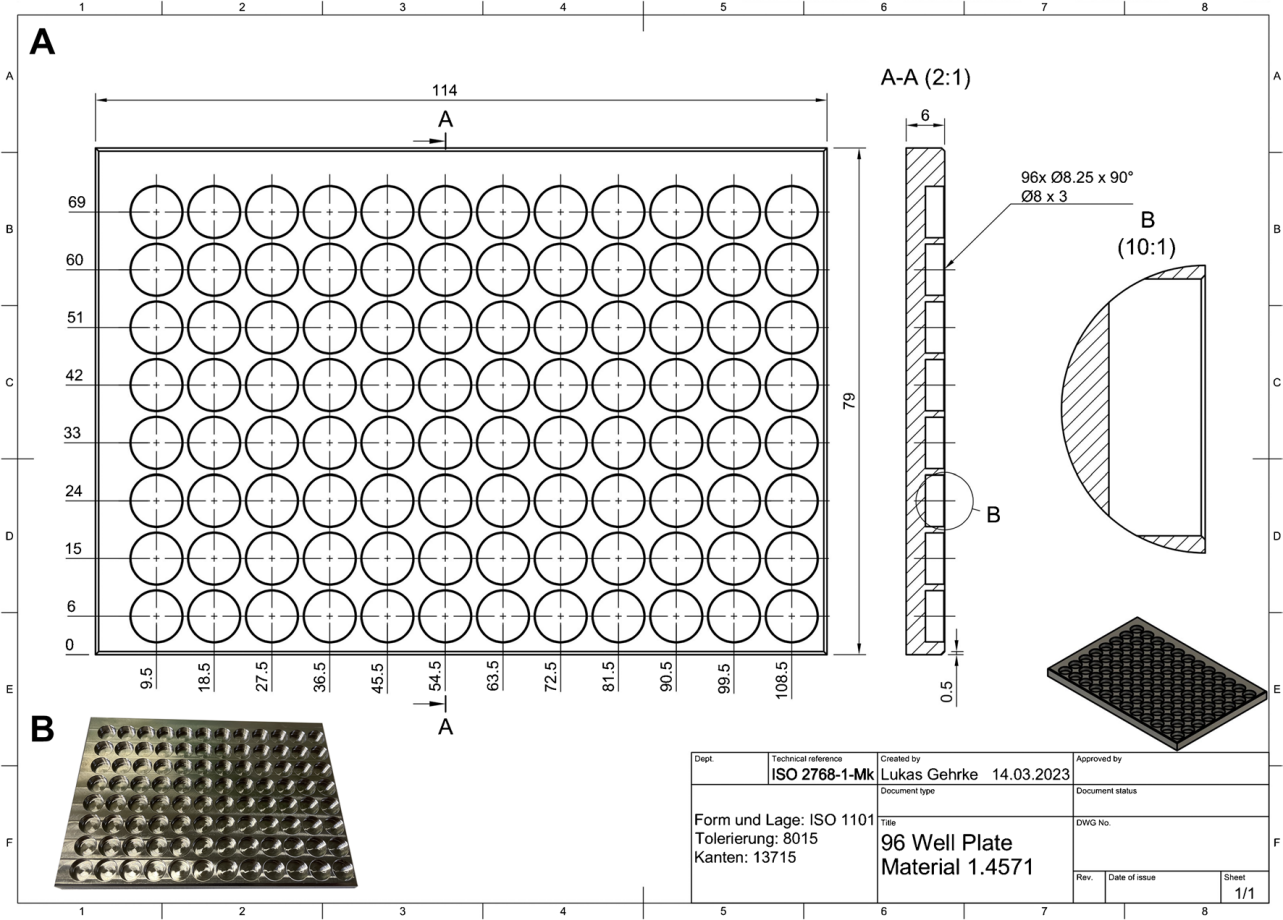
A long-term storage setup for particles from the plate reader using a metal 96-well plate. The well positions can be labeled along the left and top axis in the blank space. **A** Blueprints for the creation of the 96-well plates. **B** An image of the 96-well plate made from stainless steel. Placing particles in this plate allows for easy transfer to the FTIR plate reader plates when needed by maintaining the reference positions, and since it is made of nonplastic materials, it has low contamination risk for plastic pollution research

Reflection and transmission plates were expensive, so we fabricated nonplastic 96-well plates to hold the particles long-term. Metal 96-well plates were fabricated in-house in the scientific workshop of the Alfred Wegener Institute in corresponding positions to where they would be in the reflection or transmission plates for the plate readers (Fig. 4). The plates were stored face up in covered glass Petri dishes (Ø 18 cm), which prevent the loss of the particles from blowing wind. Storage in this way allowed all particles to be rapidly transferred to a reflection or transmission plate and reanalyzed if needed. The total time for transferring 95 particles from one plate to another was less than 15 min. Alternatively, additional reflection or glass plates could be purchased and used for long-term storage.

### Validation statistics

The technique was validated for its spectral quality by comparing the spectra collected with the Open Specy library [22], a collection of several open-access spectral databases for FTIR [4, 25]. Out-of-the-box accuracy was tested using the Open Specy package [36] and several other data cleaning and visualization packages [37–43] in R [44] with the default settings for smoothing (Savitzky–Golay filter with 11 points and a 3rd-order polynomial) [45], baseline correction (imodpolyfit 8th order polynomial) [46], and correlation (Pearson). Unknown materials were not used in assessing the validity of the library. The identification was said to be accurate if the top match returned by Open Specy was identical to the known identity of the material. The correlation values were used to infer the rationale behind lower hit qualities for some spectral collection modes. A hit quality threshold was not used to calculate out-of-the-box accuracy.

## Results and discussion

### Validation of technique

**Fig. 5:**
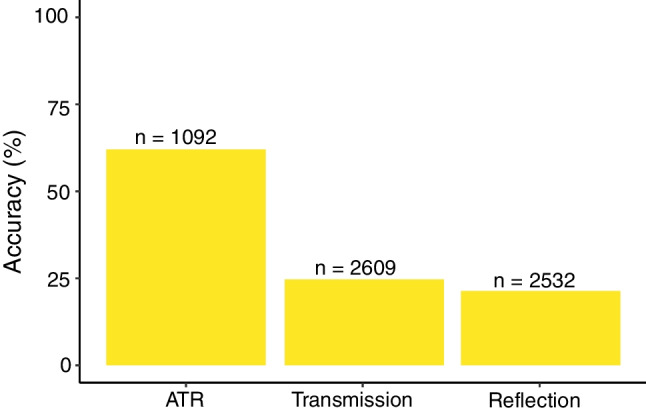
Validation of the database produced using Open Specy’s out-of-the-box settings to identify the material type. The *X*-axis is the spectral collection mode employed in collecting the database. The *Y*-axis is the accuracy in percent of correct identifications of Open Specy in identifying spectra from the spectral collection mode group. The total number of spectra tested for each spectral collection mode is listed above the bars. The height of the bars is the accuracy (%). Spectra counts were not identical across the techniques because not all particles were measured in all modes, and some particles were measured more times than others

Out-of-the-box accuracy for Open Specy in identifying the spectra we collected was best for ATR Spectra (62%), followed by transmission (25%) and reflection (21%) (Fig. 5). A similar relationship was found when only looking at particles for which we had all three spectral collection modes (Figure S1). This was unsurprising to us since Open Specy’s library primarily consisted of ATR spectra (as do most commercial products [23]), and ATR spectra can be quite different from transmission and reflection spectra. A careful user would likely achieve higher accuracy using Open Specy than out-of-the-box accuracy by counting correct “unknown” IDs as accurate IDs and manipulating the parameters in Open Specy to improve baseline subtraction and smoothing.

**Fig. 6:**
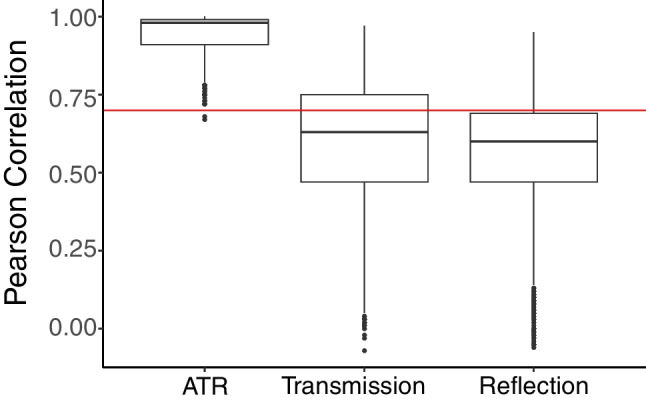
Box plots for Pearson correlation coefficient for ATR, transmission, and reflection analysis, showing the superior performance of ATR due to the current contents of the Open Specy library. The *X*-axis is the spectral collection mode, while the *Y*-axis is the mean of the maximum correlation values to the Open Specy library for all replicates of each particle’s spectra. The red horizontal line demarked 0.7 correlation below which identifications are considered uncertain. The plot shows boxplots for the maximum correlation for each spectral collection mode to the library in Open Specy. Points on the plot show outliers. Edges of the box are the inter quartile range. The center line in the box is the median

Pearson correlations for the ATR spectra were higher than 0.7 in most measurements (Fig. 6). This is likely because Open Specy primarily contains ATR spectra, with some of the previous materials characterization by ATR and reference spectra existing in the Open Specy library [4]. Transmission and reflection spectra had similarly low top hit qualities, with most spectra having a top hit below 0.7. We recommend extensive expansion to spectral libraries to include spectra from reflection and transmission spectral collection modes and to include more reference standards. Correlation values for reflection and transmission spectra were mostly below the recommended threshold of 0.7 (Fig. 6). These observations were similar if we only assessed particles with data from all three spectral collection modes (Figure S2). We recommend declaring hits below 0.7 as “unknown materials.”

**Fig. 7:**
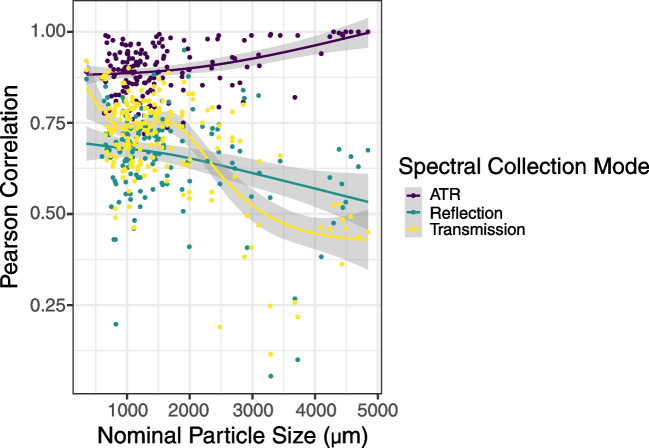
The Pearson correlation ATR, Reflection, and Transmission as a function of nominal particle size, showing an increasing correlation with increasing nominal particle size for ATR analysis. The *X*-axis is the nominal particle size (square root of particle projected area). The *Y*-axis is the mean of the maximum correlation values to the Open Specy library for all replicates of each particle’s spectra. Smooth lines are generalized additive models with a smoothing spline and 95% confidence intervals shown in gray. ATR, Reflection, and Transmission for each particle are colored differently and shown in the legend. ATR showed better correlations for larger particles, while transmission and reflection showed the opposite

Some of the variability in hit qualities was likely due to particle characteristics interfering with signal collection, such as particle size (Fig. 7). ATR correlation values increased for larger particles. We noticed that spectral hits decreased in general for the largest particles in reflection and transmission modes, likely due to higher levels of signal absorption for the larger particles. Particles with a nominal size of ~ 3 mm or less appeared to have similar maximum hits to the Open Specy library. One way to overcome this problem in the future is to make the particles as thin as possible (in the direction of the infrared beam). Although we only assessed particles down to 500 µm nominal size, it is possible that smaller particles could also be characterized using this technique; there is a lack of a strong particle size-dependent decrease in the top hit for the smallest particles tested.

### Comparing techniques

**Fig. 8:**
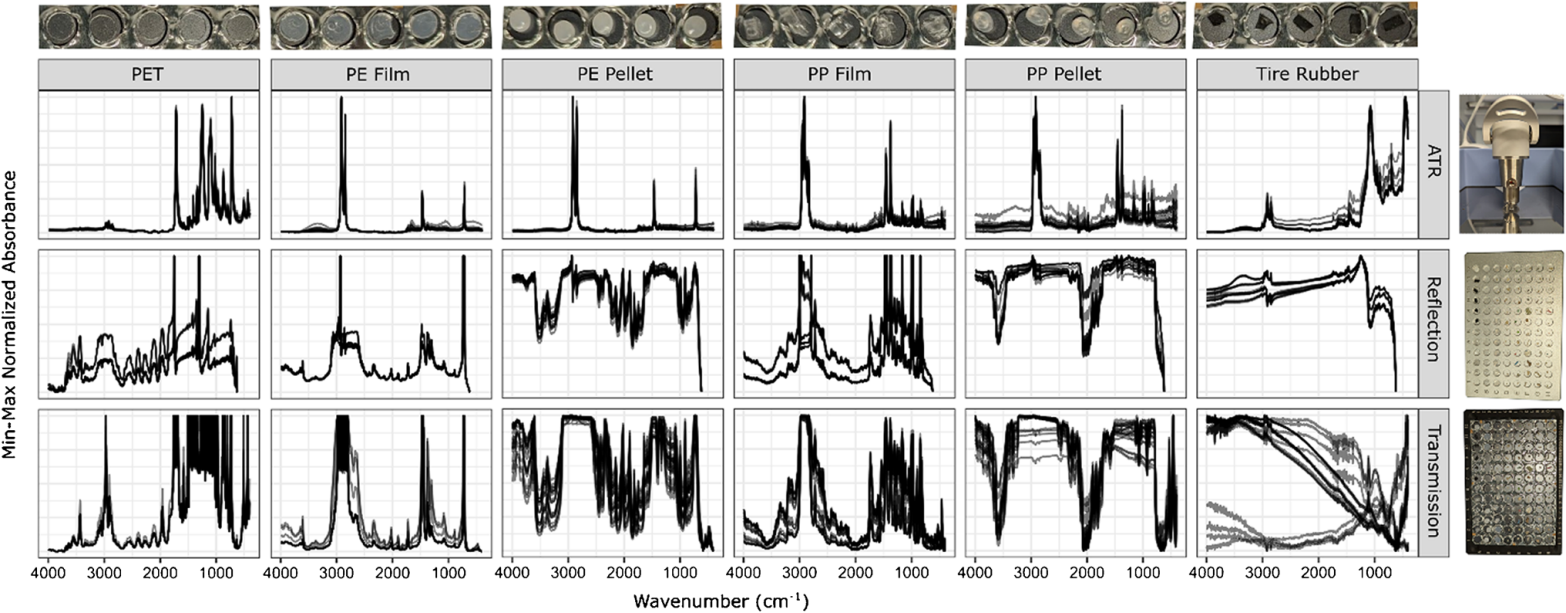
Comparison of spectra from the same materials (top axis) for each spectral collection mode (right axis). The *Y*-axis (unitless) is min–max normalized absorbance intensity values for each spectrum. The *X*-axis is wavenumbers in units cm^−1^. When multiple spectra were collected in a single mode, they are overlaid. Images on the right axis show the spectral collection modes. Images on the top axis show the particles that were assessed

Comparing the spectra acquired between ATR, reflection, and transmission, we see that all three techniques could provide similar quality spectra with similar peaks under ideal scenarios like film plastic spectra (Fig. 8, PE Film). In some cases, transmission and reflection spectra had additional strong peaks that ATR did not (Fig. 8, PET). Film plastic materials had different signals than their pellet counterpart of the same polymer type (Fig. 8, PE and PP). In ATR, this difference was not large, but the difference for reflection and transmission was drastic with the pellet counterparts having very poor spectral quality. This was likely due to the particle thickness, which can impact the signal strength of transmission and reflection (Fig. 7). Although the reflection and transmission spectra of pellets were poor, the spectra did not reach total absorbance and still have peaks that could be indicative of the material. Other differences between the signals include derivative-like distortions [47] of reflection spectra (Fig. 8, PE pellet) and a relative positive shift in absorbance intensity towards lower wavelengths for ATR of thicker samples [48] (Fig. 8). Spectral reproducibility was tested in the transmission plate reader by rerunning the same plate with slightly different settings (wavenumber resolution of 8 cm^−1^ and only one measurement of the spectra), and the particle spectra had a correlation of 0.92, indicating high reproducibility of measurements in the plate reader. Sometimes, one technique produced drastically more variable spectra than the other two for a given particle. For example, the tire rubber transmission spectra had inverse baseline signals from run to run (Fig. 8, tire rubber transmission). The shape and form of transmission and reflection spectra appear more similar than ATR spectra, suggesting that the two could be used complementarily in reference libraries (Fig. 8, PE film reflection and transmission). A known fundamental difference in the techniques is that ATR collects spectra of a thin surface of the material, while transmission and reflection techniques have deeper penetration which can change the relative intensities of peaks [48] and collect signals through polymer composite materials [49]. Regardless of spectral collection mode, we observe that spectra are generally consistent across all 5 particle runs for each material and the 3–4 replicate spectra collected per particle (Fig. 8). This was promising for using plate readers to characterize plastic particles.

The primary advantage of the plate reader method is increased speed for analyzing large microplastic and macroplastic particles compared to ATR. Based on our work with these techniques, we estimate the plate reader technique takes 1 min per particle, on average, to prepare the sample; this could then rapidly be reassessed with any number of spectral collection parameters. This method is an order of magnitude faster than ATR, typically 10 min per particle, and must be manually redone if a mistake is made. Although all these techniques are generally considered non-destructive, there were cases where particles were altered using ATR from the force of the press, or particles had to be cut to use in the plate reader. In a few cases, particles were geometrically complex and rigid, preventing us from collecting a high-quality ATR spectrum, but transmission and reflection were not impacted. There were a few cases where the particle got extremely close to the edge of the well or became sandwiched between the cover and the silicon (Fig. 2). Still, we found no evidence of particles leaving the wells in the measurements or spilling over into another well. When it is critical not to alter the particle and to collect a good-quality spectrum, great care must be taken to assess which technique is most appropriate.

## Conclusions

We presented a new technique for analyzing large microplastic and macroplastic FTIR signatures in reflection and transmission modes and compared it to traditional ATR measurement. FTIR plate readers provide higher throughput analysis of large microplastics and macroplastic samples than ATR. Using plate readers could improve accessibility to microplastic research, which can be hampered by access to expensive equipment due to long queues for instrument time to collect spectral data. The spectra acquired in transmission and reflection modes from plate readers were of sufficient quality for spectral analysis but were substantially different from ATR spectra commonly available in spectral reference libraries. We provide one of the largest and most extended open-access spectral libraries to date to accelerate the adoption of this technique. We created an off-the-shelf plate cover for transmission plate readers to keep particles in position, which could be improved in future studies if a walled well plate design was developed or a rigid metal cover was fabricated to fit the silicon plates. Last, we demonstrated that out-of-the-box identification is inappropriate for accurate spectral characterization and propose that better automated routines for spectral analysis continue to be advanced.

### Supplementary Information

Below is the link to the electronic supplementary material.

Supplementary file1 (DOCX 130 KB)

## Data Availability

Data and source code come with a CC BY NC license, allowing copying and reuse for non-commercial purposes. Commercial licenses may be sought by contacting the corresponding authors. Raw data, source code, method videos, and spectral database developed in this manuscript are available DOI: 10.5281/zenodo.10126851.
