# Supplementary material for: Generation of macro- and microplastic databases by high-throughput FTIR analysis with microplate readers
Source: Anal Bioanal Chem. 2024 Jan 13;416(6):1311–20. doi: 10.1007/s00216-024-05127-w (PMC10861755; doi:10.1007/s00216-024-05127-w)
Supplement: Supplementary file 1 — Supplementary file1 (DOCX 130 KB) [file 216_2024_5127_MOESM1_ESM.docx]

# Supplemental Information


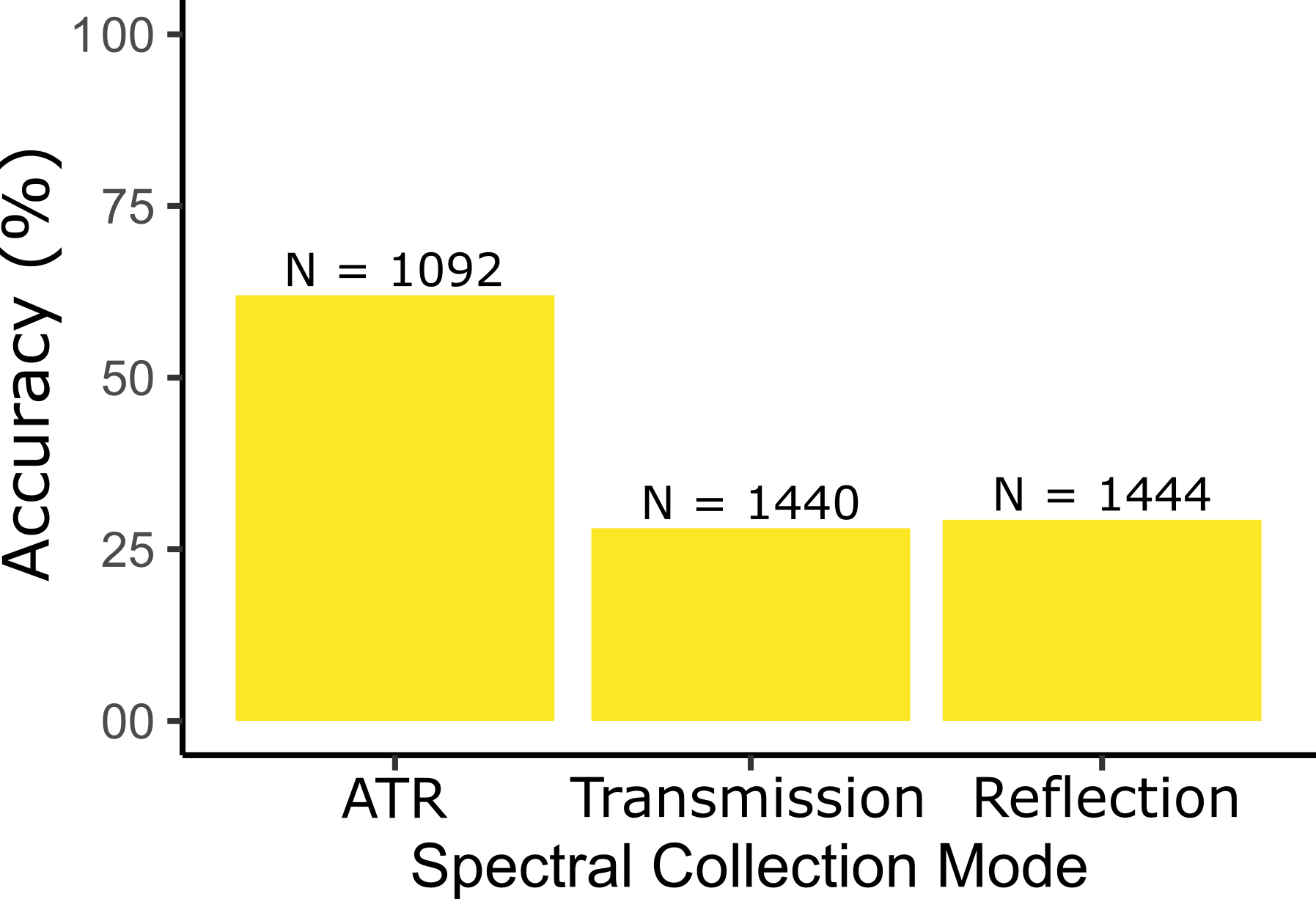


Figure S1: Complementary to a figure in the main text this one includes only particles which have spectral examples from all three spectral collection modes. Validation of the database produced using Open Specy’s out-of-the-box settings to identify the material type. X axis was the spectral collection mode employed in collecting the database. Y axis was the accuracy in percent of correct identifications of Open Specy in identifying spectra from the spectral collection mode group. The total number of spectra tested for each spectral collection mode was listed above the bars. The height of the bars was the accuracy. Spectra counts were not identical across the techniques because some particles were measured more times than others.


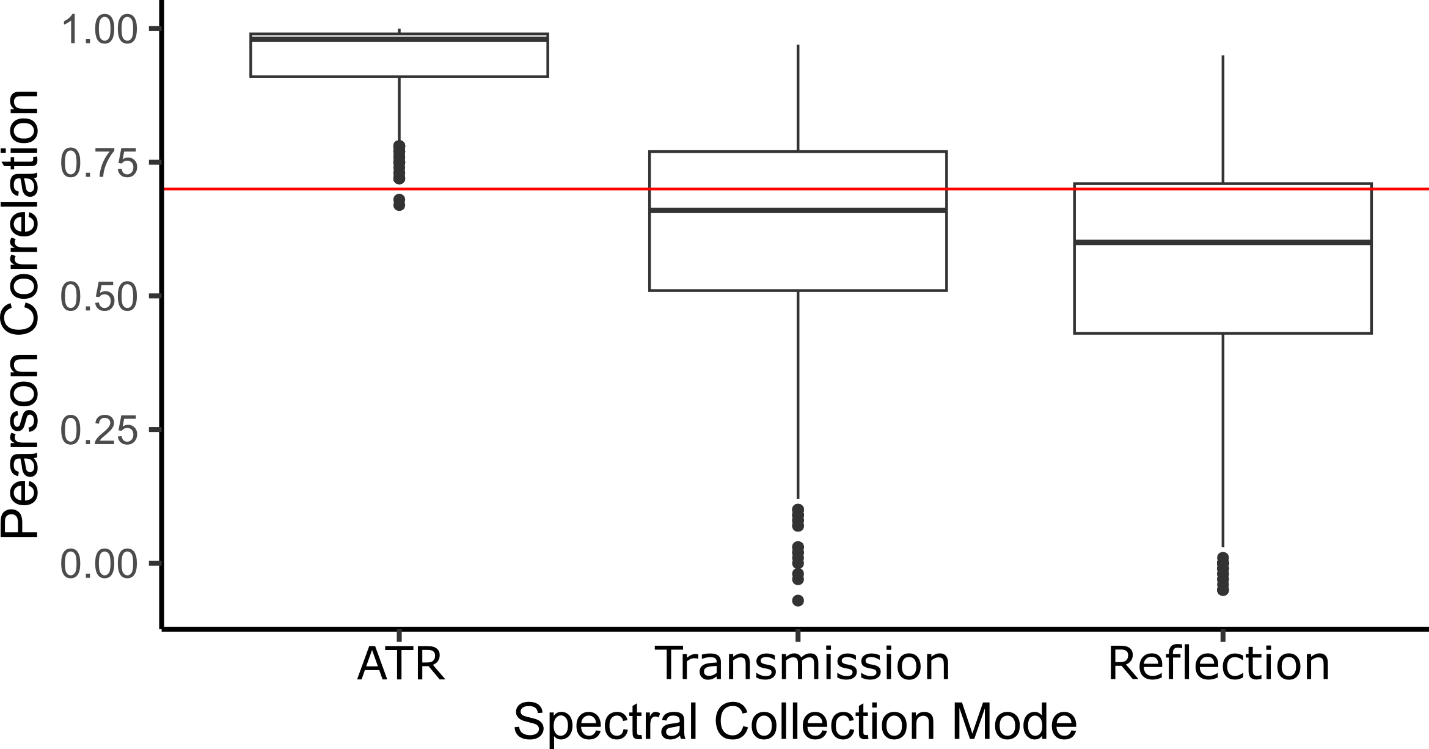


Figure S2: Complementary to a figure in the main text this one includes only particles which have spectral examples from all three spectral collection modes. X axis is the spectral collection mode. Y axis is the mean of the maximum correlation values to the Open Specy library for all replicates of each particle’s spectra. The red horizontal line marks 0.7 correlation below which identifications are considered uncertain. The plot shows boxplots for the maximum correlation for each spectral collection mode to the library in Open Specy. Points on the plot show outliers. Edges of the box are the inter quartile range. Center line in the box is the median.
